# Assessment of reporting quality in randomized controlled trials of acupuncture for labor pain

**DOI:** 10.3389/fpain.2022.999162

**Published:** 2022-11-21

**Authors:** Tao Jiang, ShiYi Jiang, Ying Cui, Ji-Peng Yang, Yuan-Hao Du, Jing Li, Bo Pang, Bo Li

**Affiliations:** ^1^ National Clinical Research Center for Chinese Medicine Acupuncture and Moxibustion, Tianjin, China; ^2^ Department of Acupuncture and Moxibustion, First Teaching Hospital of Tianjin University of Traditional Chinese Medicine, Tianjin, China; ^3^ Evidence-based Medicine Center, Tianjin University of Traditional Chinese Medicine, Tianjin, China

**Keywords:** labor pain, RCTs, acupuncture, CONSORT, STRICTA

## Abstract

**Objective:**

To evaluate the reporting quality of randomized controlled trials (RCTs) of acupuncture for labor pain, and to explore relevant factors for facilitating reporting transparency and integrity for future RCTs.

**Method:**

Eight Chinese and English databases were systematically searched from their inception until August 31, 2021. General characteristics and methodological quality of the included reports were evaluated based on the CONSORT statement and the STRICTA guidelines. Descriptive statistical analysis was performed. Cohen's *κ*-statistics were calculated to assess the agreement of all items between two reviewers.

**Results:**

A total of 84 RCTs were included. Based on the CONSORT statement, a positive reporting rate (greater than 80%) was evident for the items “trial design” “participants” “intervention” “outcomes” “numbers analyzed” and “generalizability”. The quality of reporting for the items “randomized in the title or abstract” “sample size” “allocation concealment” “implementation” “blinding” “recruitment” “ancillary analyses” “harms” “interpretation” “registration” and “protocol” was poor with positive rates less than 10%. Based on the STRICTA guidelines, the items “extent to which treatment varied” “number of needle insertions per subject per session” and “control or comparator interventions” had poor reporting quality with positive rates of less than 10%. Substantial agreement was observed for most items and excellent agreement for some items.

**Conclusion:**

The reporting quality of RCTs of acupuncture for labor pain is suboptimal generally. Rigorous adherence to the CONSORT statement and the STRICTA guidelines should be emphasized in future studies to improve the quality of acupuncture RCT reports.

## Introduction

Labor pain is one of the severe pains caused by uterine contractions, cervical dilatation, and vaginal and pelvic floor stretching (1). The pain of labor is not as well localized as somatic pain. It is diffuse and may reach the iliac crests, buttocks, or thighs. This pain adversely affects uterine oxygen consumption as well as uterine contractility, and it increases peripheral resistance, cardiac output, and blood pressure (2). Pain, anxiety, and stress during delivery increase the release of catecholamines and cortisol into circulation. Elevated cortisol levels will lead to decreased uterine blood flow and delayed contractions even the death of the newborn (3–5). Due to the increased demand for pain management, neuraxial labor analgesia is regarded as the most effective treatment for labor pain (6, 7). However, drugs used for epidural analgesia may temporarily lower blood pressure, thus reducing the blood flow to the fetus and slowing its heart rate.

Acupuncture is effective in treating various pains and has been valued and recognized in Western countries in recent years. Acupuncture is the most used treatment for pain, and it is the most widely covered by health insurance in the World Health Organization (WHO) traditional medicine strategy 2014–2023 (8). The literature on acupuncture for labor pain has grown year by year over the past two decades. Ramnero et al. (9) conducted a randomized trial of acupuncture during delivery and found that acupuncture significantly reduced the need for epidural analgesia and resulted in greater maternal relaxation when compared to a control group. However, no studies have explored the quality of the RCT reports, especially the details of the acupuncture intervention. The number of RCT reports has increased because many clinical studies on acupuncture for labor pain have been conducted both nationally and internationally (10). The distinction between Chinese and Western acupuncture results in disparate acupuncture reports. Chinese acupuncture is guided by the basic theories of Traditional Chinese Medicine, using acupuncture points as stimulation points, and emphasizing “de qi” to treat various pains such as labor pain, and regulate the functions of the whole body and internal organs. On the other hand, Western acupuncture is based on modern neuroscience knowledge and determines the trigger point and the depth of stimulation based on theories such as local axonal reflex, dorsal root reflex, homo- and cross-segmental neuromodulation, and central regulation. Western acupuncture also focuses on quantifying stimulation parameters, such as stimulation duration and the number of needles used to treat painful diseases (11, 12).

The CONSORT statement is an evidence-based, minimum set of recommendations for reporting randomized trials (13). The STRICTA recommendations comprised a checklist that expanded the generic content of item 4 of the CONSORT statement, which is set for clinical trials with controlled groups of acupuncture to report interventions. They offered a standard method for facilitating transparent and more complete reporting of acupuncture RCTs and assisting authors' critical appraisal and interpretation (14). The release of the CONSORT statement and the STRICTA guidelines positively impacted the quality of reporting (15). Both have been widely accepted as the standard for clinical trial reporting, with statistically significant increases in citations (16, 17).

To our knowledge, no study has previously investigated the quality of reporting of trials in this area against these standards. Therefore, the CONSORT statement and the STRICTA guidelines were used to evaluate the quality of reporting of RCTs of acupuncture for labor pain. We also aimed to provide authors with more favorable information for high-quality RCT designs for reference.

## Methods

### Search strategy

The following eight databases were systematically searched: Chinese National Knowledge Infrastructure (CNKI), VIP information (VIP), Wanfang Data, SinoMed, PubMed, Web of Science, Cochrane Library, and Embase, from their inception until August 31, 2021. The details of the search strategy can be found in the online Supplementary File 1. No language restrictions were applied in the search strategy.

### Inclusion and exclusion criteria

RCTs that examined the effects of acupuncture interventions for labor pain were included. The inclusion criteria are as follows.

#### Types of reports

Only RCTs were included in our research. Non-randomized trials, cross-over clinical trials, case-control studies, retrospective studies, animal experiments, case reports, and reviews were excluded. RCT test reports without available data or results were also excluded.

#### Participants of included RCTs

All study subjects were women aged 21 to 31 years old, including primipara and multipara, with a normal singleton pregnancy and a fetus in a cephalic presentation at a gestational age of 37 to 42 weeks, at 3–6 cm cervical dilation of labor, and without any obstetrical complications for vaginal delivery. Patients must give verbal or written consent to participate in the study. Patients with hypertension, diabetes mellitus, and coronary heart disease were excluded because these diseases can have an impact on delivery, with a significantly higher incidence of surgical delivery, birth injuries, and postpartum hemorrhage which would affect the trial.

#### Types of interventions

Acupuncture is manual or electronic stimulation due to filiform needle penetration of the body, scalp, or auricular acupoints regardless of diameter, length, manufacturer, or material. The study group included any type of invasive acupuncture, including manual acupuncture, electroacupuncture, or auricular (ear) acupuncture, or in combination with other interventions (e.g., acupuncture-related treatment, drugs, physical therapy) were included. Trials that compared the effectiveness of acupuncture therapy with sham acupuncture or active control procedures were included. Studies testing non-filiform-needle-penetration (e.g., acupoint injection, bleeding with plum-blossom needles) as a primary intervention were excluded. Our study did not include studies comparing non-invasive techniques such as laser acupuncture or acupressure or moxibustion trials. The control groups included the use of placebo, treatment, as usual, no treatment, or other active interventions. Regarding the control interventions, RCTs using Chinese herbal medicine were excluded because of the ambiguous pharmacological mechanism and curative effect. Moreover, trials that only compared different forms of acupuncture were also excluded since we did not intend to investigate whether one type of acupuncture was more effective than another.

#### Types of outcome measures

In our review, the assessment of acupuncture for labor pain focused on authoritative indicators are as follows: (1) CONSORT score status (2) STRICTA score status (3) Cohen's *κ*-statistical analysis, and 95% CIs for each report.

### Document screening

Two researchers independently searched the Chinese and English databases (Shi-Yi Jiang for PubMed, EMBASE, Cochrane, and Web of Science; Ying Cui for CNKI, VIP, Wanfang, and SinoMed), and the third reviewer (Tao Jiang) organized the searched articles using the EndNote X9 software. Duplicate records were initially identified and removed by the reviewer (Li Jing) using EndNote X9.

After removing duplicates, two reviewers (Shi-Yi Jiang and Ying Cui) independently evaluated the articles for relevance by title and abstract to look for potentially eligible studies. Subsequently, read the full texts for potential inclusion. If an article did not meet the inclusion criteria and/or met one or more exclusion criteria, it was moved to the folder marked “excluded” in EndNote X9. Some controversial articles were marked into the “questionable” folder, in which case they were resolved through discussion and consensus among the three reviewers. The third reviewer (Tao Jiang) also verified all the information and contacted the primary authors for unavailable articles if needed.

### Data extraction

Three reviewers (Shi-Yi Jiang, Ying Cui, and Ji-Peng Yang) extracted the information from each included trial into predefined data collection forms meeting the Cochrane standard. In the “characteristics of trials” form, studies were described in terms of author, country, participants, interventions, control types, frequency and treatment course, duration of one session, and main outcomes. The two reviewers resolved the situation through discussion and negotiation if the data could not be determined during the extraction process. If needed, we contacted the trial's lead author by email to provide incomplete data. In addition, “data extraction” forms were used to record and calculate relevant data for the outcomes.

### Assessment of reporting quality

Two reviewers (Shi-Yi Jiang and Ying Cui) used the CONSORT statement and STRICTA guidelines to assess the reporting quality of the included RCTs independently. Each item was scored 1 if it was reported and 0 if it was not clearly stated. Any difficulties or disagreements in the process were solved by the third reviewer (Tao Jiang).

Cohen's *κ*-statistic was calculated to evaluate the degree of agreement between the two evaluators. A *κ* of 0.40 or lower, between 0.40 and 0.60, between 0.60 and 0.80, and from 0.80 to 1.00 were considered poor, moderate, substantial, and perfect agreements, respectively. The number, percentage, Cohen's *κ*-statistical analysis, and 95% CIs of each variable were summarized using the Statistical Package for the Social Sciences (SPSS) V 26.0.

## Results

**Figure 1:**
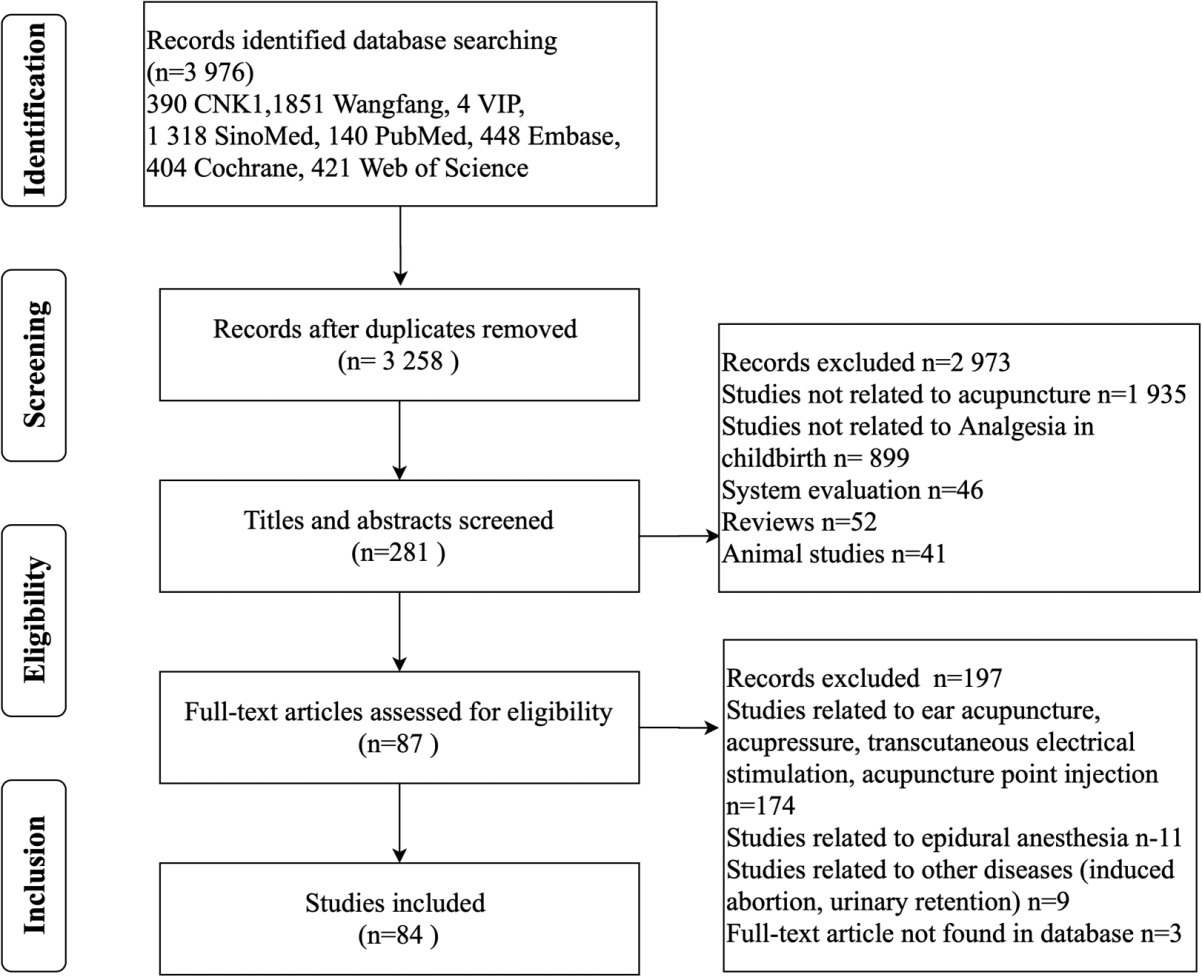
Process and results of literature screening.

The initial search identified a total of 3, 976 relevant reports, of which 2, 563 were published in Chinese and 1, 413 were published in English. After eliminating duplicates, 3, 258 research articles were left for further consideration. After screening titles and abstracts, 2, 977 records were excluded due to relevance to the topic of interest, leaving 281 studies to be evaluated for inclusion. Articles involving acupressure, patching, transcutaneous electrical stimulation, acupoint injections, epidural anesthesia, and ear acupuncture were excluded. Articles, where the original article could not be found, were also excluded. The final 84 eligible RCTs were extracted for analysis. The search and selection process is outlined in Figure 1.

### Study characteristics

#### Years of publication

**Figure 2:**
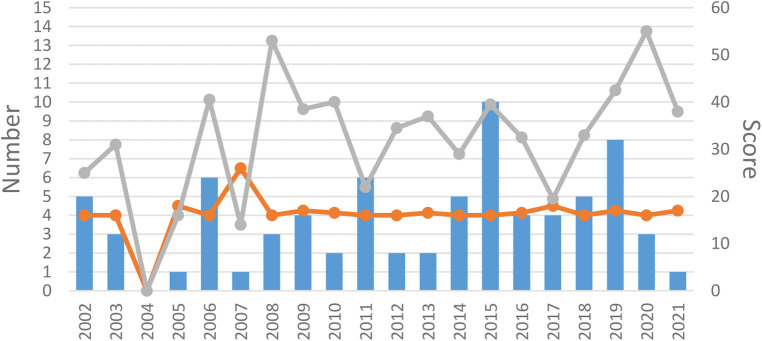
Year of publication. The blue bar is the number of RCTs published each year. The orange line is the median OQS with CONSORT statement of each year. The gray line is the median OQS with STRICTA statement of each year.

All 84 articles were RCTs published from 1974 to 2021. 31(36.9%) RCTs were published in the early period (before 2010), and 53 (63.1%) RCTs were in the late period (after 2010). All included articles were in Chinese (73.8%) or English (26.2%). We can find the number of publications of RCTs after 2010 is about double the number before 2010. The trend graph for the year of publication is outlined in Figure 2.

#### Journals and language

These 84 papers were published in 63 different journals. 3 (3.5%) were published in the Shanghai Journal of Acupuncture and Moxibustion, 2 (2.3%) in the Chinese Journal of Acupuncture and Moxibustion, and 3 (3.5%) in the Journal of Clinical Acupuncture and Moxibustion. Among the 87 RCT papers, 62 (73.8%) were written in Chinese. The remaining 22 (26.2%) were published in English and appeared in 18 different journals. In terms of journal type, 58 (69%) were published in general medical journals, 13 (15.5%) in specialty medical journals, and 13 (15.5%) in traditional or alternative medical journals.

#### Participants

12, 014 full-term deliveries including primipara and multipara, were recruited in 84 RCTs, with sample sizes ranging from 12 to 500. Participants were recruited as outpatients or inpatients in a hospital setting. All participants had to meet the required delivery conditions, with no contraindications to obstetric delivery, no serious comorbidities, complications, etc. All trials restricted the age range and medication use of participants. In addition, all the included trials excluded structural abnormalities.

#### Intervention/controls and comparison

Interventions include manual acupuncture or electroacupuncture alone, acupuncture combined with medication, or other interventions such as relaxation techniques. The most used acupuncture points are LI-4 and SP-6 mainly. 61 of these trials required the presence of a “De qi” sensation after stimulation, which is the key factor in the effectiveness of acupuncture treatment. The controls in the trials were generally used as blank controls, and only eight trials used sham acupuncture as a control group.

#### Outcome measures

Of the trials of acupuncture for labor pain, 41 assessed pain using the visual analog scale (VAS), 11 trials used the WHO pain scale for assessment, 38 trials measured labor time, 28 trials assessed bleeding at 2 h postpartum, and 27 trials used the Apgar scores.

### Reporting quality evaluation

#### Reporting quality based on CONSORT

The ratings of reporting quality based on the CONSORT statements are listed in Table 1. The positive reporting rates of items such as “trial design” “participants” “intervention” “outcomes” and “outcomes and estimation” were above 80%. However, the quality of reporting in items “randomized in the title or abstract” “sample size” “allocation concealment” “implementation” “blinding” “recruitment” “intent-to-treat analysis” “ancillary analyses” “limits” and “trial protocol” was very poor with positive rates <10%. According to the statistical results, there were no reports describing the following relevant items: “description of the similarity of interventions if relevant” “methods for additional analyses, such as subgroup analyses and adjusted analyses” “dates deﬁning the periods of recruitment and follow-ups” “subgroup analysis and adjusted analysis, distinguishing the pre-speciﬁed from the exploratory” “all important adverse or unintended effects in each group”. In this study, Cohen's *κ*-statistic showed that both reviewers agreed on all items. Consistency was judged to be moderate, substantial, or perfect for most items.

**Table 1:** Assessment of reporting quality using items from the CONSORT statement (*n* = 84studies).

Criteria	Item	Description	Number of Positive trials	% (*n*/84)	Cohen's *к*	95% CI
Title and abstract						
	1a	Identiﬁcation as a randomized trial in the title.	3	3.57	1.00	0.05–0.08
	1b	Structured summary of trial design, method, results, and conclusions (for speciﬁc guidance see CONSORT for abstracts).	84	100	1.00	1.00
Introduction						
Background and objectives	2a	Scientiﬁc background and explanation of rationale.	16	19.05	0.88	0.11–0.28
	2b	Speciﬁc objectives or hypotheses.	77	91.67	0.39	0.86–0.98
Methods						
Trial design	3a	Description of trial design (such as parallel, factorial) including allocation ratio.	84	100.00	1.00	1.00
	3b	Important changes made to the method after the trial commencement (such as eligibility criteria), with reason.	81	96.43	0.58	0.92–1.01
Participants	4a	Eligibility criteria for participants.	83	98.81	0.39	0.96–1.01
	4b	Settings and locations where the data were collected.	73	86.90	1.00	0.80–0.94
Interventions	5	Sufﬁcient details of each intervention, including how and when they were administered to allow replication.	76	90.48	0.82	0.84–0.97
Outcomes	6a	Deﬁned pre-speciﬁed primary and secondary outcome measures, including how and when they were assessed.	77	91.67	0.62	0.86–0.98
	6b	Any changes to trial outcomes after the beginning of the trial, and the reasons.	79	94.05	0.64	0.89–0.99
Sample size	7a	How was the sample size determined.	82	97.62	0.66	0.94–1.01
	7b	Explanation of any interim analyses and stopping guidelines when applicable.	3	3.57	0.02	0.05–0.08
Randomization						
Sequence generation	8a	Methods used to generate the random allocation sequence.	8	9.52	0.94	0.03–0.16
	8b	Type of randomization; details of any restrictions (such as blocking and block size).	80	95.24	0.71	0.91–1.00
Allocation concealment mechanism	9	The mechanism used to implement the random allocation sequence (such as sequentially numbered containers), description of any steps taken to conceal the sequence until the assignment of interventions.	8	9.52	0.84	0.03–0.16
Implementation	10	Who had generated the random allocation sequence, who had enrolled participants, and who had assigned participants to the interventions.	3	3.57	0.02	0.05–0.08
Blinding	11a	Who had been blinded (for example, participants, care providers, those assessing outcomes) and how.	3	3.57	0.02	0.05–0.08
	11b	Description of the similarity of interventions if relevant.	0	0.00	1.00	0.00–0.01
Statistical methods	12a	Statistical methods used to compare the primary and secondary outcomes in each group.	82	97.62	0.42	0.94–1.01
	12b	Methods for additional analyses, such as subgroup analyses and adjusted analyses.	0	0.00	1.00	0.00–0.01
Results						
Participant flow (a diagram is strongly recommended)	13a	For each group, the numbers of randomly assigned participants, whether they received the intended treatment, and whether the primary outcome was analyzed.	82	97.62	0.56	0.94–1.01
	13b	Losses and exclusions after randomization in each group and the corresponding reasons.	5	5.95	0.82	0.01–0.11
Recruitment	14a	Dates deﬁning the periods of recruitment and follow-ups.	0	0.00	1.00	0.00–0.01
	14b	The reason why the trial was stopped or terminated.	1	1.19	0.66	0.01–0.04
Baseline data	15	A table showing baseline demographics and clinical characteristics for each group.	11	13.10	1.00	0.06–0.21
Numbers analyzed	16	For each group, the number of participants (denominator) included in each analysis and whether the analysis had been performed by the originally assigned groups.	80	95.24	0.53	0.91–1.00
Outcomes and estimation	17a	The primary and secondary outcomes result for each group, the estimated effect size and its precision (such as 95% conﬁdence interval).	81	96.43	0.42	0.92–1.01
	17b	The presentation of both absolute and relative effect sizes was recommended for binary outcomes.	6	7.14	0.63	0.02–0.13
Ancillary analyses	18	Results of any other performed analysis, including subgroup analysis and adjusted analysis, distinguishing the pre-speciﬁed from the exploratory.	0	0.00	1.00	0.00–0.01
Harms	19	All important adverse or unintended effects in each group (for speciﬁc guidelines see CONSORT for adverse effects)	0	0.00	1.00	0.00–0.01
Discussion						
Limitations	20	Trial limitations, addressing sources of potential bias, imprecision, and, if relevant, multiplicity of analyses	10	11.90	0.77	0.05–0.19
Generalizability	21	Generalizability (external validity, applicability) of the trial ﬁndings	84	100.00	0.65	1.00
Interpretation	22	Interpretation consistent with results, balancing beneﬁts and side effects, and considering other relevant evidence	6	7.14	0.78	0.02–0.13
Other information						
Registration	23	Registration number and name of trial registry	2	2.38	1.00	0.01–0.06
Protocol	24	Where the full trial protocol can be accessed, if available	1	1.19	1.00	0.01–0.04
Funding	25	Sources of funding or other supports (such as drug supply), role of funders	14	16.67	1.00	0.09–0.25

#### Reporting quality based on STRICTA

The reporting quality ratings for the needling details based on the STRICTA guidelines are listed in Table 2. Good reporting existed for the items “style of acupuncture” “names of points used” “needle stimulation” and “explanation to patients”, with positive ratings >80%. However, the reporting quality for items “extent to which treatment varied” “number of needle insertions per subject per session” and “control or comparator interventions” was poor, with positive rates <10%. Cohen's *κ*-statistic showed that good agreement was observed for the items “needle stimulation” “number of treatment sessions” and “control or comparator interventions”. Perfect agreement was observed for the other remaining items.

**Table 2:** Assessment of reporting quality of needling details from STRICTA (*n* = 84 studies).

Criteria	Item	Description	Number of Positive trials	% (n/84)	Cohen's *к*	95% CI
Acupuncture rationale (explanations and examples)	1a	Style of acupuncture (eg, Traditional Chinese Medicine, Japanese, Korean, Western medical, Five Element, ear acupuncture).	80	95.24	1.00	0.91–1.00
	1b	Reasoning for provided treatment, based on historical context, literature sources, or consensus methods, with references where appropriate.	15	17.86	0.96	0.10–0.26
	1c	Extent to which treatment varied.	0	0	1.00	0.00–0.01
Details of needling (explanations and examples)	2a	Number of needle insertions per subject per session (mean and range where relevant).	5	5.95	0.88	0.01–0.11
	2b	Names (or location if there was no standard name) of points used (uni/bilateral).	81	96.43	1.00	0.92–1.01
	2c	Depth of insertion, based on a speciﬁed unit of measurement, or on a particular tissue level.	25	29.76	0.91	0.20–0.40
	2d	Response sought (eg, de qi or muscle twitch response).	61	72.62	1.00	0.63–0.82
	2e	Needle stimulation (eg, manual, electrical).	75	89.29	0.75	0.83–0.96
	2f	Needle retention time.	37	44.05	0.88	0.33–0.55
	2g	Needle type (diameter, length, and manufacturer or material).	32	38.10	0.95	0.28–0.49
Treatment regimen (Explanations and examples)	3a	Number of treatment sessions.	25	29.76	0.78	0.20–0.40
	3b	Frequency and duration of treatment sessions	23	27.38	0.91	0.18–0.37
Other components of treatment (explanations and examples)	4a	Details of other interventions administered to the acupuncture group (eg, moxibustion, cupping, herbs, exercises, lifestyle change).	25	29.76	0.91	0.20–0.40
	4b	Setting and context of treatment, including instructions to practitioners, information, and explanations to patients.	84	100	1.00	1.00
Practitioner background (explanations and examples)	5	Description of participating acupuncturists (qualiﬁcations or professional afﬁliations, years in acupuncture practice, other relevant experiences).	40	47.62	0.95	0.37–0.59
Control or comparator interventions (explanations and examples)	6a	The rationale for the control or comparator in the context of the research question, with sources that justify this choice.	4	4.76	0.71	0.00–0.09
	6b	A precise description of the control or comparator. If sham acupuncture or any other type of acupuncture-like control was used, provide details as in items 1–3 above.	7	8.33	0.82	0.02–0.14

## Discussion

This study first systematically assessed and analyzed the quality of reported RCTs of acupuncture for labor pain by statistical methods, adhering strictly to the CONSORT statement and STRICTA guidelines. It has been demonstrated that RCTs of acupuncture for labor pain were generally varied in reporting quality in the past two decades, with a considerable number of essential items incomplete or omitted. This may substantially bias readers' judgment of the actual and verifiable results of research, as well as their external validity (18, 19).

### CONSORT statement

Generally, scores of reporting quality were not satisfactory enough in the 84 trials in accordance with the CONSORT statement, with only 16 of the total 37 items (including sub-items of each section) showing relatively sufficient reporting rates (above 85%). The under-reported items are particularly those concerned methodological sections such as methods of generating random sequence, allocation concealment and implementation of randomization, as well as blinding. This is consistent with results of our previous review on the annual evidence of reported RCTs using acupuncture and moxibustion therapy during 2019 and 2020, which found an insufficiency in reporting of randomization and blinding (20).

Regarding the randomization section, it is suggested that authors should provide sufficient information so that the reader can evaluate the likelihood of bias in the process of generating random allocation sequence and grouping (item 8a) (13). However, it was merely reported by eight trials, of which only one trial fully reported methods of sequence generation and allocation concealment. Besides, for the description of the type of randomization (item 8b), though reported in 95.24% of the research, most trials only referred to “random” “random number tables” and “opaque envelopes”; Some studies, despite the use of the word “random”, have used non-random or quasi-random methods, such as “alternating grouping” “grouping by hospital number”. Moreover, how the random sequence is implemented is important when the subject enters a trial (item 9), and the ideal approach in this case is to use allocation (21), aiming to prevent selection bias (22). While only eight of the included trials mentioned in their reports of using sequentially numbered, opaque, sealed envelopes, none had reported if envelopes are opened in order and other details. Such flaws in reporting of detailed randomization process will impact the bias risk assessment and subsequently, the judgment of research reliability and adaptability (18, 23).

Blinding, additionally, is used for preventing bias in implementation and outcome determination (21), for the purpose of protecting random sequences after the allocation occurs. However, blinding is not always possible. Since the late 1970s, clinical trials have been using a “real vs. sham” model to evaluate the effects of acupuncture (24), with much effort devoted to the development and validation of “sham” (or designated as “placebo” by some researchers) needling, for the points of view of minimizing therapeutic effects and successful blinding (25). To date, however, there still lacks consensus on the complete inertness of either non-penetrating needling appliance (such as Streitberger needle, Park sham device, Takakura sham device) or “sham” penetrating process (e.g., needling at non-specific points, shallow insertion, minimal stimuli), as well as their reliability for blinding of acupuncturists or patients (26). The true effect size of acupuncture may be greatly underestimated when compared to that of the current sham controls (26, 27). Besides, the recognition rate of two types of sham needling procedures (either shallow penetrating or needling at non-specific points) has been shown as high as 50% to 83% among health volunteers (28); the blinded effect of non-penetrating devices was also not ideal, especially among subjects with acupuncture experience (29). According to our findings, only three included trials briefly mentioned the use of blinding—all performed on the patients. In general, such issues of blinding and sham controlling in acupuncture trials still need to be addressed to promote their feasibility and reporting quality.

Another issue is the determination of the sample size, which was only 3.57% of the included trials. The lack of a basis for determining the sample size calculation is an important factor restricting clinical research; inaccurate calculation will also cause the waste of effort and resources and impact the quality of research (30). Moreover, in terms of additional information, the International Committee of Medical Journal Editors (ICMJE) requires all clinical trials to be registered to improve transparency and accountability (31). However, only two trials reported the clinical trial registration numbers on ClinicalTrials.gov, and one published the full protocol. However, most RCTs did not provide a complete trial protocol and did not mention funding sources and other support. This is not conducive to the standardized management of clinical studies and the quality of clinical trials. Both authors and editors need to pay attention to it (32).

### STRICTA guidelines

The STRICTA guidelines was developed in 2010 as a formal extension of the CONSORT statement and had become an independent guideline for regulating the details of the acupuncture procedure (33). It has been found that half of the published articles reviewed lacked intervention elements and insufficient detail, and journals have increased their requirements for the use of this guideline in recent years (34). However, contradictions exposed between trials largely reflected the discrepant processing relevant to ethnic and environmental differences, culture, policies, as well as physician's skills, and correspondingly, the variant “therapeutical habits” in acupuncture procedure (frequency, course, needling site, manipulation, time for needle retention, et al.) (35, 36). The STRICTA checklist assessment in our study indicated moderate or low reporting quality with most items, especially explanations of needling, treatment regimen and controls.

First, reporting rates for the number of needles and the needling depth in Chinese articles were much lower than English publications. Chinese acupuncturists tend to judge the therapeutic effect by patients' status or the “*deqi*” responses and apply individual regimen *via* targeted point selection and manipulation. While Western (or called modern) acupuncture relatively focuses on the trigger-point theory and quantitative parameters during stimulation (37, 38). Despite the difference, inadequate reporting of items or details of the manipulation is a barrier to future replication. Related studies have shown that the number of needles inserted and the depth of needle insertion, as well as the correct acupuncture point, can be crucial to the effectiveness of acupuncture treatment (34), providing a valuable reference for clinicians. Moreover, items related to contextual factors and practitioner qualifications were also severely lacking, which will impact the validity and reliability of those interventions.

In addition, there showed a difference in the use of controls between trials. Most of studies conducted in China applied no-treatment or drug controls, while in Western studies, up to 36% used sham acupuncture controls. Control groups for acupuncture should also be rigorously described to facilitate other researchers seeking to replicate acupuncture control interventions, assess the internal validity of clinical outcomes, and promote the use of effective interventions in clinical practice (39, 40). This is particularly important for empirical medicine like acupuncture, as different acupuncturists treating different study subjects may affect the generalizability of trial results. Therefore, details of the interventions for the experimental and control groups need to be explained in a structured format. In practice, acupuncture is a practitioner-dependent, experience-requiring intervention. These items meant that under-reporting of items or details related to manipulation and technique was a significant barrier to future replication studies. Therefore, full compliance with STRICTA guidelines is emphasized in the design, implementation, and reporting of acupuncture RCTs to ensure the generalizability and reliability of studies.

In the evaluated RCTs, no articles reported adverse effects, possibly because the adverse effects were not taken seriously or were not documented. The reporting of adverse events, especially for invasive interventions, is critical to understanding safety issues. The human body can have varying degrees of adverse reactions to any intervention, ranging from nausea, vomiting, bruising, and bleeding in mild cases to pneumothorax and even death in severe cases.

Despite the strict inclusion criteria and methodology, this study still has some limitations. Due to language limitations, we only included Chinese and English reports of RCTs in this area; countries and regions with high rates of acupuncture use, like Japan and Korea, are not included. Both researchers inserted subjective opinions in the CONSORT and STRICTA quality assessment of each included RCT, and although we used Cohen's *κ*-statistic to reduce inconsistency between evaluators, this still does not exclude bias in the results due to subjective evaluations. Publication bias caused by journal editors, authors, funding sources, and literature inclusion is a serious problem in systematic reviews and meta-analyses, which can affect the validity and generalization of conclusions. Most of the articles included in the journal were dominated by positive results, while negative results may be delayed in publication or ignored, resulting in publication bias (41).

## Conclusion

In conclusion, this study indicates that the quality of RCTs of acupuncture for maternal labor pain was inadequate and needs further improvement, especially in terms of some key methodological entries and acupuncture details. Future RCTs still need further refinement under the CONSORT statement and STRICTA guidelines as both have become important references for journals and editors in evaluating and selecting manuscripts. Researchers are recommended to adopt such guidelines when designing or reporting acupuncture RCTs to enhance the reporting quality and transparency of their studies.

## Data Availability

The original contributions presented in the study are included in the article/**Supplementary Material**, further inquiries can be directed to the corresponding author/s.
