# Supplementary material for: Assessment of reporting quality in randomized controlled trials of acupuncture for labor pain
Source: Front Pain Res (Lausanne). 2022 Nov 21;3:999162. doi: 10.3389/fpain.2022.999162 (PMC9720146; doi:10.3389/fpain.2022.999162)
Supplement: Supplementary file 1 [file Datasheet1.docx]

**Search strategy**

The search strategy for PubMed

((acupuncture[MeSH Terms]) OR (acupuncture therapy[MeSH Terms]) OR (electroacupuncture[MeSH Terms])OR (acupuncture point[MeSH Terms])) AND ((labor[MeSH Terms]) OR (childbirth[MeSH Terms]) OR (delivery[MeSH Terms]) OR (parturition[MeSH Terms])) AND ((pain[MeSH Terms]) OR (ache[MeSH Terms]) OR (analgesia[MeSH Terms]))

The search strategy for Web of science

TS=(acupuncture* OR acupuncture therapy* OR electroacupuncture* OR acupuncture point* OR acupoint)AND TS=(childbirth* OR labor* OR delivery* OR parturition)AND TS=(pain* OR ache* OR analgesia)

The search strategy for Embase

('acupuncture'/exp OR 'acupuncture therapy'/exp OR 'electroacupuncture'/exp OR 'needling'/exp OR 'acupuncture point'/exp OR 'acupoint'/exp) AND ('labour'/exp OR 'delivery'/exp OR 'parturition'/exp OR 'childbirth'/exp) AND ('pain'/exp OR 'ache' OR 'analgesia'/exp)

The search strategy for CNKI

((SU = '针灸' OR SU = '针刺' OR SU = '电针'OR SU = '针' OR SU = '刺' OR SU = '穴' OR SU = '穴位' ) AND (SU = '妊娠' OR SU = '生产' OR SU = '产中' OR SU = '分娩' OR SU = '分娩期') AND (SU = '疼痛' OR SU = '痛' OR SU = '镇痛')) AND (FT = '随机')

The search strategy for Wanfang

主题:((“针灸” or “针刺” or “电针” or “针” or “刺”or “穴位”) and (“妊娠” or “生产” or “产时” or “分娩” or “分娩期”) and ("疼痛" or “痛” or “镇痛”)) and 全部:(“随机”)

The search strategy for VIP

M=(针灸 OR 针刺 OR 电针 OR 针 OR 刺 ) AND M=(分娩 OR 分娩期 OR 妊娠 OR 生产 OR 产中 ) AND M=(疼痛 OR 痛 OR 镇痛) AND U=随机

The search strategy for SinoMed

(("针灸"[常用字段:智能] OR "针刺"[常用字段:智能] OR "电针"[常用字段:智能] ) AND ("分娩"[常用字段:智能] OR "分娩期"[常用字段:智能] OR "妊娠"[常用字段:智能] OR "生产"[常用字段:智能] OR "产中"[常用字段:智能] ) AND ("疼痛"[常用字段:智能] OR "痛"[常用字段:智能] OR "镇痛"[常用字段:智能])) AND "随机"[全部字段:智能]
